# Supplementary material for: Optical Gain of Vertically Coupled Cd0.6Zn0.4Te/ZnTe Quantum Dots
Source: Nanomaterials (Basel). 2023 Feb 13;13(4):716. doi: 10.3390/nano13040716 (PMC9965561; doi:10.3390/nano13040716)
Supplement: Supplementary file 1 [file nanomaterials-13-00716-s001.zip › nanomaterials-2193963-supplementary.pdf]

Supporting information

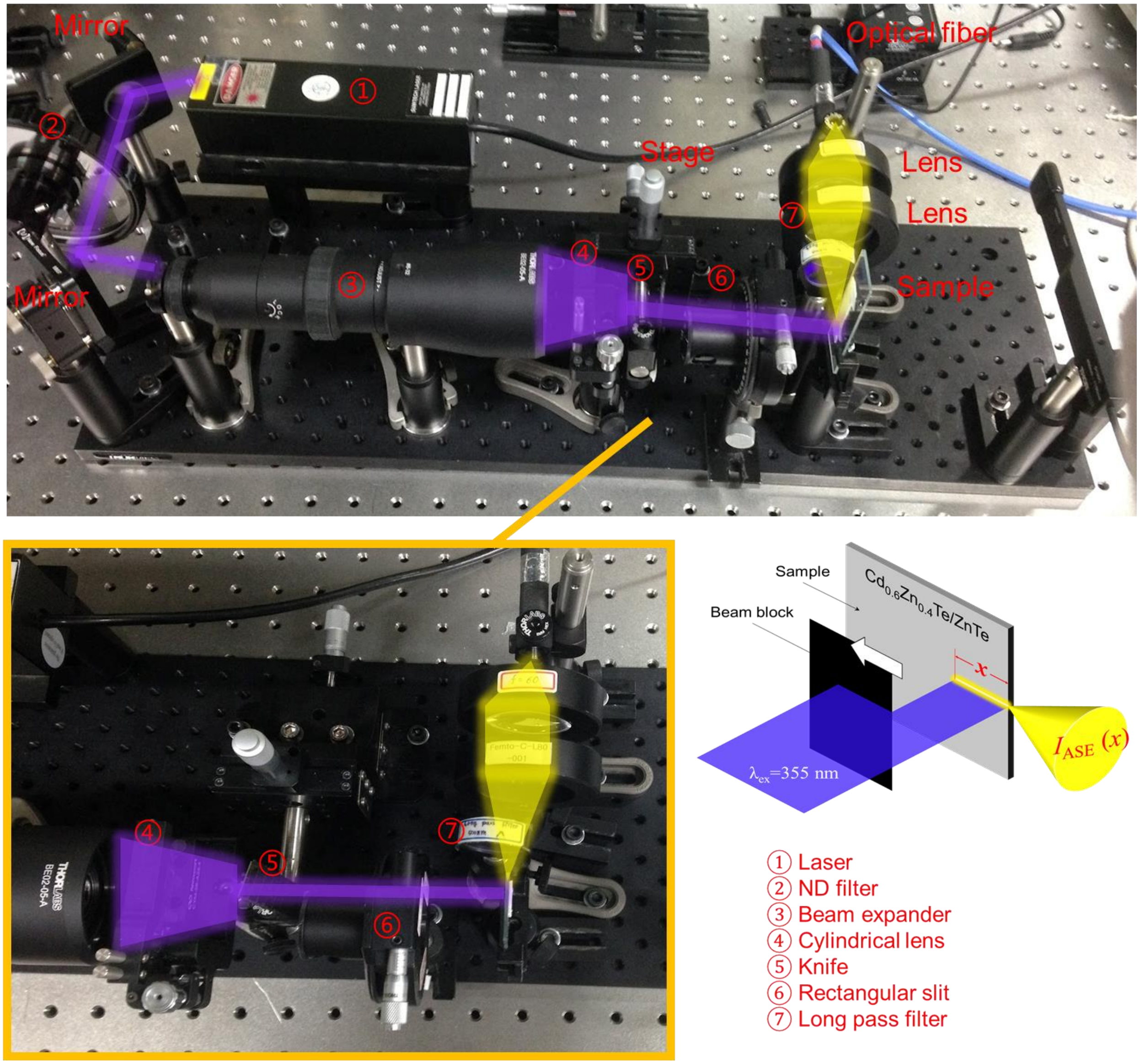

Figure S1. Optical alignment of the variable stripe length method

Optical alignment of gain measurement was shown in Figure S1 as well as a simplified schematics of variable stripe length method. For excitation, a nanosecond pulsed laser ① (355 nm) with 20 kHz repetition rate was utilized to excite sample, and ND filter ② was used to change excitation intensity. In order to prepare a homogeneous light intensity of beam stripe, a beam expander ③ was utilized, whereby the beam diameter becomes increased. This beam becomes focused by a cylindrical lens ④ to make a stripe shape, and a knife ⑤ was utilized to vary the beam stripe length (x). Since the incident laser spot contains the Gaussian distribution, the optical stripe still contains an intensity inhomogeneity. However, the beam expander also increases the size of an optical stripe. Using a rectangular slit ⑥, the uniform intensity area can be selected, and the optical stripe becomes rectangular. We found the diffraction effect is negligible. Finally, a nice rectangular optical stripe can be prepared on sample. In order to check the beam uniformity,

the optical stripe image can be measured directly by an image sensor. Alternatively, a knife-edge method can be used to measure the intensity distribution.

For room temperature gain measurement, the optimum condition can be obtained. When sample is mounted in an extra cryostat for low temperature gain, all optical distances should be changed regarding the size of cryostat.

To collect the edge emission of amplified spontaneous emission, a long-pass filter ⑦ is necessary to cut laser light, and a pair of lens are used to introduce the ASE to detector. For convenience, an optical fiber can be used to detect ASE. In this case, the numerical aperture should be considered to find the suitable focal distance of the detection lens. For low temperature gain measurement of vertically coupled quantum dots, we used a monochromator, where CCD camera is attached.
